# Identification of characteristics of foot position and angle during swing phase in fallers using principal component analysis

**DOI:** 10.3389/fbioe.2023.1117884

**Published:** 2023-02-14

**Authors:** Takuma Inai, Yoshiyuki Kobayashi, Chenhui Huang, Koji Fujita, Masahiro Fujimoto, Fumiyuki Nihey, Akiko Yamamoto, Kanako Nakajima, Kentaro Nakahara, Gaku Kutsuzawa, Kenichiro Fukushi, Shoma Kudo

**Affiliations:** ^1^ QOL and Materials Research Group, National Institute of Advanced Industrial Science and Technology, Tokyo, Japan; ^2^ Exercise Motivation and Physical Function Augmentation Research Team, National Institute of Advanced Industrial Science and Technology, Tokyo, Japan; ^3^ Biometrics Research Labs, NEC Corporation, Tokyo, Japan; ^4^ Department of Functional Joint Anatomy, Graduate School of Medical and Dental Sciences, Tokyo Medical and Dental University, Tokyo, Japan; ^5^ Department of Orthopaedic and Spinal Surgery, Graduate School of Medical and Dental Sciences, Tokyo Medical and Dental University, Tokyo, Japan

**Keywords:** fallers, gait, foot, position, swing phase, principal component analysis

## Abstract

Identifying the characteristics of fallers is important for preventing falls because such events may reduce quality of life. It has been reported that several variables related to foot positions and angles during gait (e.g., sagittal foot angle and minimum toe clearance) differ between fallers and non-fallers. However, examining such representative discrete variables may not be sufficient to detect crucial information, which may be contained in the large portions of unanalyzed data. Therefore, we aimed to identify the comprehensive characteristics of foot position and angle during the swing phase of gait in non-fallers and fallers using principal component analysis (PCA). Thirty non-fallers and 30 fallers were recruited for this study. We performed PCA to reduce the dimensions of foot positions and angles during the swing phase and obtained principal component scores (PCSs) for each principal component vector (PCV), which were then compared between groups. The results revealed that the PCS of PCV3 in fallers was significantly larger than that in non-fallers (*p* = 0.003, Cohen’s d = 0.80). We reconstructed waveforms of foot positions and angles during the swing phase using PCV3 and our main findings can be summarized as follows. Compared to non-fallers, fallers have a 1) low average foot position in the *z*-axis (i.e., height) during the initial swing phase 2) small average foot angle in the *x*-axis (i.e., rotation in the sagittal plane), during the initial swing phase, and 3) large variability in foot position in the *y*-axis (i.e., anterior/posterior position) during the initial swing phase. We can conclude that these are characteristics of gait related to fallers. Therefore, our findings may be beneficial for evaluating fall risk during gait using a device such as a shoe- or insole-embedded inertial measurement unit.

## 1 Introduction

Falls are a common cause of injuries such as fractures in older adults. For example, falls may cause distal radius fractures (Drinka, 1994; Meena et al., 2014; Karl et al., 2015; Rundgren et al., 2020), proximal humerus fractures (Baron et al., 1996; Karl et al., 2015), and femoral neck fractures (Drinka, 1994; Dargent-Molina et al., 1996; Yang et al., 2020), and it has been reported that these fractures reduce the ability to perform activities of daily living (Lin and Chang, 2004; Edwards et al., 2010; Fukui et al., 2012; Vergara et al., 2016). Furthermore, approximately 17%–19% of patients die within a year following hip fracture surgery (Civinini et al., 2019; Morri et al., 2019). Therefore, it is important to prevent falls in older adults and to identify the characteristics of older adults who are prone to falls.

Several previous studies have reported that some variables related to foot position and angle during gait (e.g., sagittal foot angle at heel contact (Chiba et al., 2005), minimum toe clearance (Chiba et al., 2005; Delfi et al., 2021), stride length (Kerrigan et al., 2000; Lee and Chou, 2006; Newstead et al., 2007), and variability of stride length (Reelick et al., 2011; Doi et al., 2020; Bytyçi and Henein, 2021) are significantly different between non-fallers and fallers. This knowledge is beneficial for identifying the characteristics of older adults who are prone to falls. However, previous studies (Chiba et al., 2005; Reelick et al., 2011; Doi et al., 2020; Bytyçi and Henein, 2021; Delfi et al., 2021) have focused on discrete variables of foot positions and angles during gait in older adults at risk of falling. However, if we focus only on discrete variables, it may not be possible to detect crucial information in large portions of unanalyzed data. For example, although it has been reported that large variability in lower-limb joint angles during the initial swing phase are important characteristics related to fallers compared to non-fallers (Kobayashi et al., 2014), variability in foot positions and angles during the initial swing phase has not been examined.

To resolve this issue, we propose using principal component analysis (PCA). PCA is a multivariate analysis technique that can reduce the dimensionality of data and extract principal component vectors (PCVs) and has been used to identify comprehensive characteristics of human movements in many previous studies (Maurer et al., 2012; Boudarham et al., 2013; Federolf et al., 2013; Kobayashi et al., 2014; Tsuchida et al., 2022). Therefore, using PCA, we can perform comprehensive analysis and identify novel characteristics of foot positions and angles during the swing phase in non-fallers and fallers, which has not been attempted before.

Therefore, we aimed to identify the comprehensive characteristics of foot position and angle during the swing phase of gait in non-fallers and fallers using PCA. We consider that greater balance ability is required to appropriately move the body mass from the trailing limb to the leading limb from toe-off to the initial swing phase; therefore, we hypothesize that the variability of the anterior/posterior foot position during the initial swing phase in fallers is larger compared to that in non-fallers.

## 2 Methods

### 2.1 Participants

First, we performed a *t*-test power analysis using the “pwr” package of the R language, version 4.0.2 (R Development Core Team). The significance level, power, and effect size were set to 0.05, 0.8, and 0.8 (large) (Cohen, 1992), respectively. As a result, approximately 26 participants for each group were required. Therefore, we recruited 30 participants for each group for this study. Sixty community-dwelling older adults were recruited for this study. The fall history of each participant was recorded prior to our experiment. Thirty participants experienced falls within 12 months prior to the experiment. Based on the experience of falls over the past year for each participant, we divided the participants into a non-faller group (*n* = 30) and faller group (*n* = 30) (30 non-fallers [age: 68.9 ( ± 3.1) years, height: 1.60 ( ± 0.08) m, body mass: 58.3 ( ± 9.5) kg, 15 females] and 30 fallers [age: 69.4 ( ± 3.3) years, height: 1.61 ( ± 0.07) m, body mass: 60.6 ( ± 8.8) kg, 15 females]). The inclusion criteria for participants were defined as follows: participants must be 1) able to walk independently without a walking aid and 2) over 65 years old. The exclusion criteria for participants were defined as follows: those with (1) orthopedic or neurological diseases, 2) pain in the lower limbs, and 3) visual impairment. According to previous studies, the effects of walking aids (Mundt et al., 2019), orthopedic diseases (e.g., osteoarthritis (Schmitt et al., 2015), neurological diseases (e.g., stroke (Boudarham et al., 2013), pain in the lower limbs (e.g., femoroacetabular impingement (Lewis et al., 2018), visual impairment (Saucedo and Yang, 2017), and age (Chehab et al., 2017) affect the kinematics of the lower limbs during gait and/or gait parameters. Therefore, these effects may also affect the foot position and angle during the swing phase. To eliminate these effects on foot position and angle during the swing phase, we established the five criteria listed above. The experimental protocol was approved by the ethics committee of the National Institute of Advanced Industrial Science and Technology (IRB number: 71120030-E−20150303-002). All participants provided written informed consent prior to the experiment.

### 2.2 Experiment

A motion capture system (VICON MX, VICON, Oxford, UK) with 15 cameras was used to capture marker trajectories at a sampling frequency of 200 Hz. Six force plates (AMTI, MA, United States) were used to obtain the ground reaction force at a sampling frequency of 1,000 Hz. Fifty-five reflective markers were attached to the body of each participant based on the guidelines of the Visual 3D software (C-Motion Inc: MD, United States) (Kobayashi et al., 2019). Then, before the walking trials, the participants were allowed sufficient practice walks to ensure that a natural gait was maintained. After the practice, all participants walked barefoot on a straight 10-m-long path in our laboratory at a comfortable speed. Five successful trials were recorded for the left leg of each participant.

### 2.3 Data analysis

#### 2.3.1 Data processing

The raw marker trajectory data were filtered using a fourth-order Butterworth filter with zero lag and a cutoff frequency of 10 Hz (Franz et al., 2015; Bakke and Besier, 2020; Hida et al., 2021). For the raw vertical ground reaction force data during gait, we reduced values of less than 20 N–0 N. The timing of the first left heel contact, toe-off, and second heel contact were detected using the vertical ground reaction force data and used to define the swing phase and calculate gait parameters (see Section 2.3.5 for additional details).

#### 2.3.2 Local coordinate system of the left foot

**FIGURE 1:**
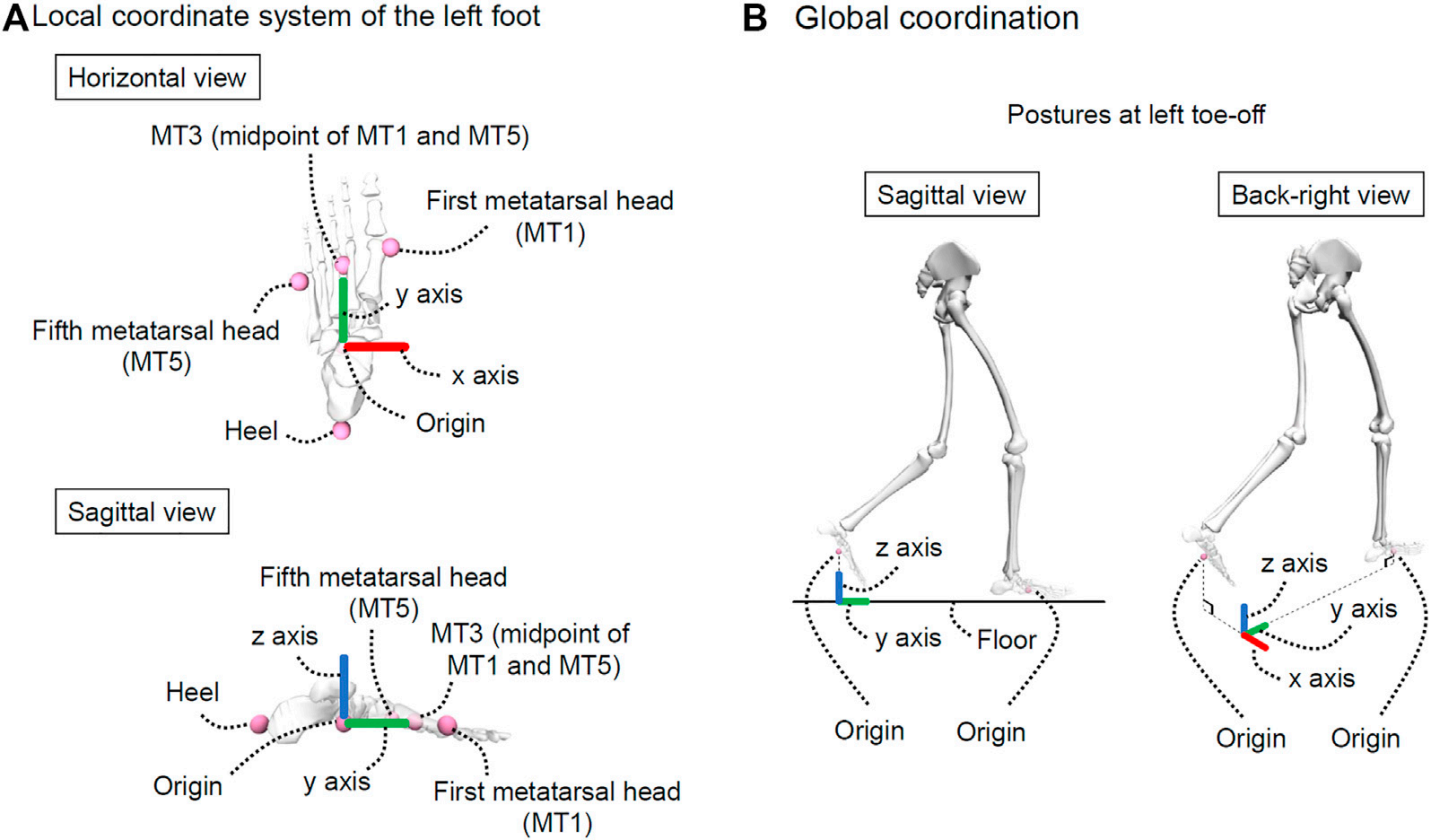
Explanation of coordinate systems. **(A)** represents the local coordinate system of the left foot and **(B)** represents the global coordination. This figure was created using OpenSim (Delp et al., 2007; Seth et al., 2018).

As shown in Figure 1A, we defined a local coordinate system for the left foot as follows.

(1) Origin: Midpoint of the left heel marker and MT3 (i.e., midpoint of the left first and fifth metatarsal head markers [MT1 and MT5, respectively]; MT3 is a virtual marker).
(2) *x*-axis (left [−]/right [+]): The unit vector is the cross product of the *y*-axis and *z*-axis.
(3) *y*-axis (posterior [−]/anterior [+]): The unit vector from the left heel marker to MT3.
(4) *z*-axis (inferior [−]/superior [+]): The unit vector is the cross product of a vector from the left heel marker to MT1 and the *y*-axis. The specific equations are as follows:

$$\vec{v_{y}}=\frac{\vec{p_{MT3}}-\vec{p_{heel}}}{\left\|\vec{p_{MT3}}-\vec{p_{heel}}\right\|}$$ (1)

$$\vec{v_{z}}=\frac{\left(\vec{p_{MT1}}-\vec{p_{heel}}\right)\times \vec{v_{y}}}{\left\|\left(\vec{p_{MT1}}-\vec{p_{heel}}\right)\times \vec{v_{y}}\right\|}$$ (2)

$$\vec{v_{x}}=\vec{v_{y}}\times \vec{v_{z}}$$ (3)

where $\vec{p_{MT3}}$ , $\vec{p_{heel}}$ , $\vec{p_{MT1}}$ , $\vec{v_{x}}$ , $\vec{v_{y}}$ , and $\vec{v_{z}}$ are the positions of left MT3, heel, and MT1, and unit vectors of the local coordinate system of the left foot, respectively.

#### 2.3.3 Foot positions and angles during the swing phase

The origin of the local coordinate system of the left foot was considered as the foot position during the swing phase. The foot positions along the *x* and *y*-axes at toe-off differed between trials. Therefore, as shown in Figure 1B, we defined another local coordinate system called the “global coordination” and used this local coordinate system to unify foot positions at toe-off. In other words, the foot positions (i.e., origin of the local coordinate system of the left foot) in the global coordination during the swing phase were calculated. The foot positions were normalized by body height for each participant because body height affects segment length (Dempster and Gaughran, 1967) and may affect foot positions during gait. According to a previous study, data in one gait cycle is generally time-normalized to 101 frames (Kobayashi et al., 2014), and the swing phase accounts for approximately 40% of one gait cycle. For this reason, the foot positions during the swing phase were time-normalized to 41 frames.

Additionally, the foot angles during the swing phase were calculated using the local coordinate system of the left foot. Specifically, the Cardan angles (sequence: x-y-z) of the local coordinate system of the left foot were calculated with respect to the global coordination. The foot angles during the swing phase were also time-normalized to 41 frames.

#### 2.3.4 PCA for foot positions and angles

**FIGURE 2:**
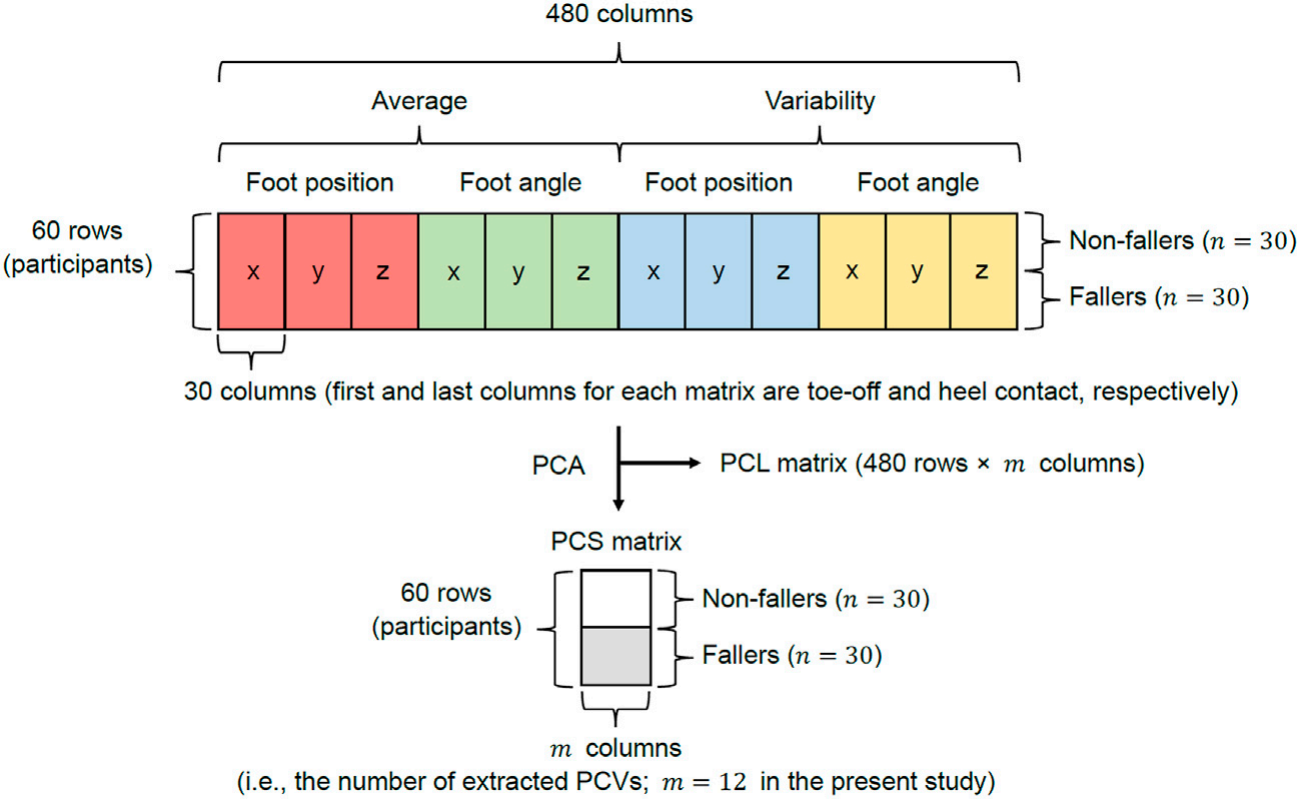
Explanation of the matrix used for PCA. The red, green, blue, and yellow matrices for each axis represent the average foot position, average foot angle, variability in foot position, and variability in foot angle, respectively.

According to a previous study (Kobayashi et al., 2014), there are differences in the averages and variabilities of kinematics during gait between non-fallers and fallers. Therefore, we also focused on the averages and variabilities of foot positions and angles during the swing phase for each participant. Subsequently, as shown in Figure 2, we obtained a 60 × 480 matrix (row: 60 [n]; column: 480 = 40 frames × 2 variables [foot positions and angles] × 3 axes [x, y, and z] × 2 types [average and variability]) and PCA was performed on this matrix. Although the foot position and angle for each axis during the swing phase were time-normalized to 41 frames, we removed the data from the first frames of the foot positions and angles. This is because the foot position on the *y*-axis in the first frame is zero, so we cannot conduct PCA if we include the first frame. Additionally, the principal component scores (PCSs) of each PCV were extracted until their cumulative contribution ratio reached 80% of the total variance. This was done because a previous study reported that a cumulative contribution ratio of 80% (or 90%) is commonly adopted to choose PCVs (Li et al., 2016). Furthermore, we compared the PCSs between the two groups (see Section 2.4 for additional details). If there is a significant difference in PCSs between non-fallers and fallers, then the foot positions and angles during the swing phase corresponding to a specific PCV can be interpreted as the characteristic foot positions and angles related to risk of falling.

Waveform reconstruction for each foot position and angle was performed based on the methods described in previous studies (Kobayashi et al., 2014, 2016; Kobayashi and Ogata, 2018; Hida et al., 2021; Tsuchida et al., 2022). In this study, the specific formulas used to reconstruct waveforms were defined as follows:

$$M_{waveform}=M_{mean}+M_{SD}M_{PCL}M_{PCS}$$ (4)

$$M_{mean}={\left[\begin{array}{ccc} m_{1} & \cdots & m_{480} \end{array}\right]}^{T}$$ (5)

$$M_{SD}=\left[\begin{array}{ccc} \sigma_{1} & \cdots & 0\\ \vdots & \ddots & \vdots \\ 0 & \cdots & \sigma_{480} \end{array}\right]$$ (6)

$$M_{PCL}=\left[\begin{array}{ccc} x_{1,1} & \cdots & x_{1,12}\\ \vdots & \ddots & \vdots \\ x_{480,1} & \cdots & x_{480,12} \end{array}\right]$$ (7)

$$M_{PCS}={\left[\begin{array}{ccc} y_{1} & \ldots & y_{12} \end{array}\right]}^{T}$$ (8)

where $M_{waveform}$ , $M_{mean}$ , $M_{SD}$ , $M_{PCL}$ , and $M_{PCS}$ are a 480 × 1 matrix of reconstructed waveforms, 480 × 1 matrix containing the mean value m for each frame, 480 × 480 diagonal matrix containing the standard deviation (SD) σ for each frame, 480 × 12 matrix containing the principal component loading (PCL) x for each frame and PCV, and a 12 × 1 matrix containing the PCS y for each PCV. To reconstruct waveforms, $y_{3}$ was set to +3 (fallers) or −3 (non-fallers) because only the PCSs of PCV3 were significantly different between groups (see Section 3 for additional details). Additionally, $y_{1}$ to $y_{12}$ were set to zero, except for $y_{3}$ . A PCL is a correlation coefficient between a PCV and a corresponding original variable, and it takes values ranging from −1 to 1. A larger absolute value of a PCL indicates a stronger correlation between the PCV and its corresponding original variable, indicating that the original variable explains the PCV well.

#### 2.3.5 Gait parameters and kinematics

According to previous studies, it has been reported that the averages and/or variabilities of gait parameters differ between non-fallers and fallers (e.g., average gait speed (Kerrigan et al., 2000; Lee and Chou, 2006; Kobayashi et al., 2014), stride length (Kerrigan et al., 2000; Lee and Chou, 2006) and variability of stride length (Reelick et al., 2011; Doi et al., 2020; Bytyçi and Henein, 2021)). Therefore, we calculated the average and variability for each gait parameter (stride time, stance time, swing time, cadence, gait speed, stride length, step width, rate of stance phase, maximum and minimum toe clearances, timing of maximum and minimum toe clearances, sagittal foot angle at toe-off, peak [negative] sagittal foot angle, sagittal foot angle at peak knee flexion angle, sagittal foot angle at heel contact, timing of peak sagittal foot angle, and peak knee flexion angle) and compared these variables between groups to help us understand gait characteristics.

Stride time was defined as the time from the first left heel contact to the second heel contact. The stance time was defined as the time from the first left heel contact to toe-off. Swing time was defined as the time from the left toe-off to the second heel contact. Cadence was calculated using stride time (i.e., cadence = 60 × 2/stride time). Gait speed (unit: m/s) was calculated from the stride time and anterior/posterior component of the reflective marker on the sacrum. The stride length was calculated as the distance along the sagittal plane between the left heel marker at the first heel contact and second heel contact. Step width was calculated as the distance in the frontal plane between the reflective markers on both heels at the second heel contact. The stride length and step width were normalized by the height of each participant (unit: m/HT) based on a previous study (Herrero-Larrea et al., 2018). The stance phase rate was calculated from the stride and swing times. The maximum toe clearance was defined as the maximum value from 0% to 60% of the swing phase and the corresponding time was also obtained. The minimum toe clearance was defined as the minimum value from 60% to 90% of the swing phase and the corresponding time was also obtained. A previous study (Yamagata et al., 2019) revealed that the toe clearance waveform is bimodal (the timing of the first maximum, minimum, and second maximum toe clearances were approximately 30%, 60%, and 90% of the swing phase, respectively). Therefore, we considered the same ranges to obtain maximum and minimum toe clearances in this study. The maximum and minimum clearances were normalized by the height of each participant (unit: mm/HT) based on a previous study (Ullauri et al., 2019). The sagittal foot angle at toe-off, peak sagittal foot angle, sagittal foot angle at peak knee flexion angle, and sagittal foot angle at second heel contact were calculated using the local coordinate system of the left foot and global coordination (Cardan angle). The timings of the peak sagittal foot angle and peak knee flexion angle were also obtained.

Additionally, we calculated the averages of the sagittal pelvis, hip, knee, and ankle angles at each time point (i.e., toe-off, peak sagittal foot angle, maximum toe clearance, peak knee flexion angle, minimum toe clearance, and heel contact) and compared these variables between groups to gain a deeper understanding of gait characteristics. All data analyses were performed using Scilab 6.1.1 (Scilab Enterprises, France).

### 2.4 Statistical analysis

We compared the PCSs, averages of gait parameters, variabilities of gait parameters, and kinematics between groups. Specifically, the Shapiro-Wilk test was used to confirm normality for each variable. The F-test was used to confirm whether two variables had the same variance. Depending on normality and variance, we compared the PCSs, averages, and variabilities of gait parameters between groups using the Student’s t-test, Welch’s *t*-test, or Wilcoxon rank-sum test. The significance level was set at *p* < 0.05. Additionally, Cohen’s d effect sizes were interpreted as small ( 0.2≤d<0.5 ), medium ( 0.5≤d<0.8 ), and large ( 0.8≤d ) based on a previous study (Cohen, 1992). Furthermore, we calculated the Pearson correlation coefficients between the PCV PCSs related to fall risk and all gait parameters. To clarify the causes of differences in the characteristics of foot positions and angles between groups, we also conducted linear regression analyses. The dependent variables were the maximum toe clearance, minimum toe clearance, sagittal foot angle at toe-off, peak sagittal foot angle, sagittal foot angle at peak knee flexion angle, and sagittal foot angle at heel contact. The independent variables were hip flexion, knee flexion, and ankle dorsiflexion angles for each timing. All statistical analyses were performed using the R language 4.0.2.

## 3 Results

**FIGURE 3:**
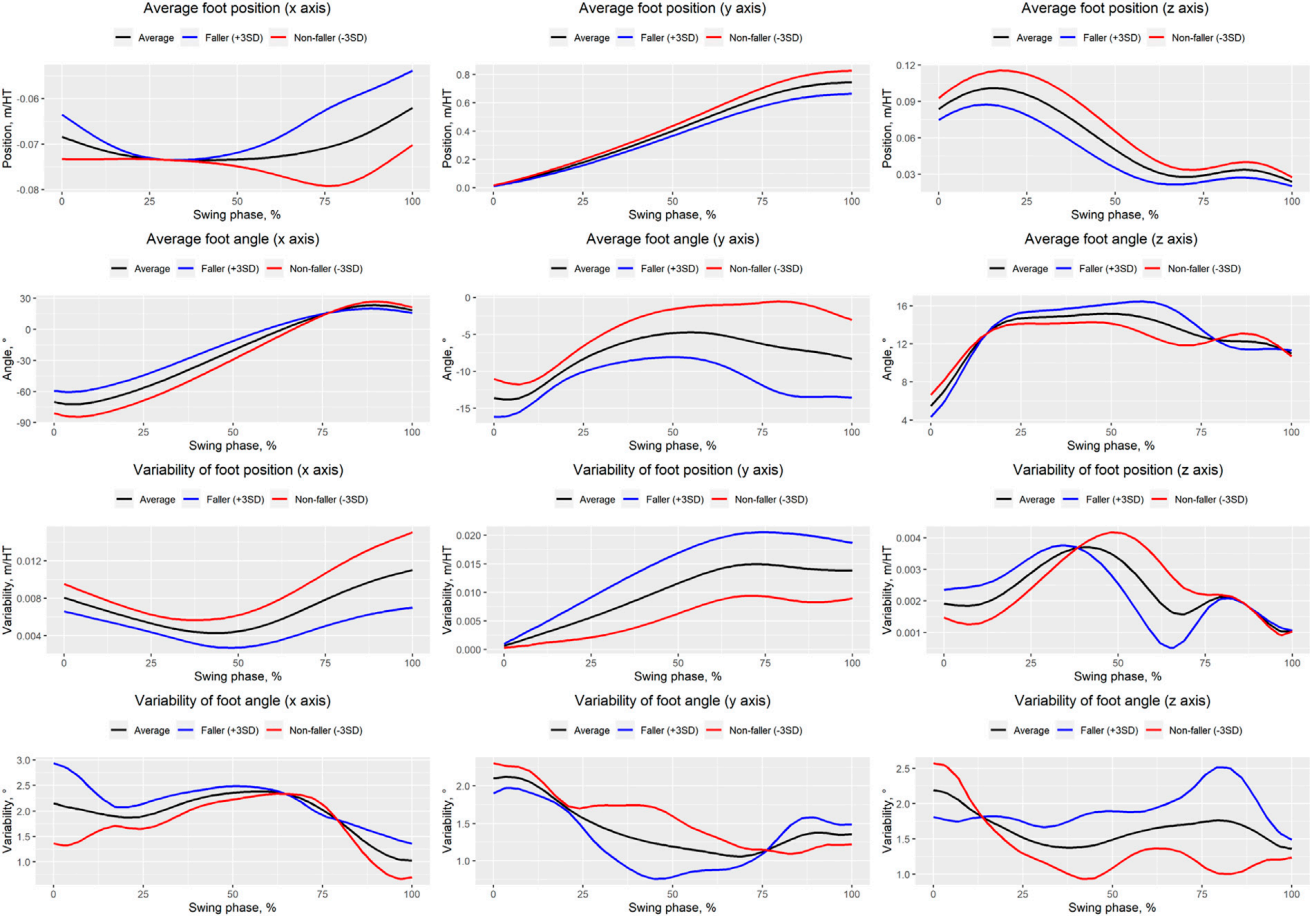
Waveforms of averages and variabilities of foot positions and angles during the swing phase. Black, blue, and red solid lines indicate the average, faller-like pattern ( + 3SD), and non-faller-like pattern (−3SD), respectively.

**FIGURE 4:**
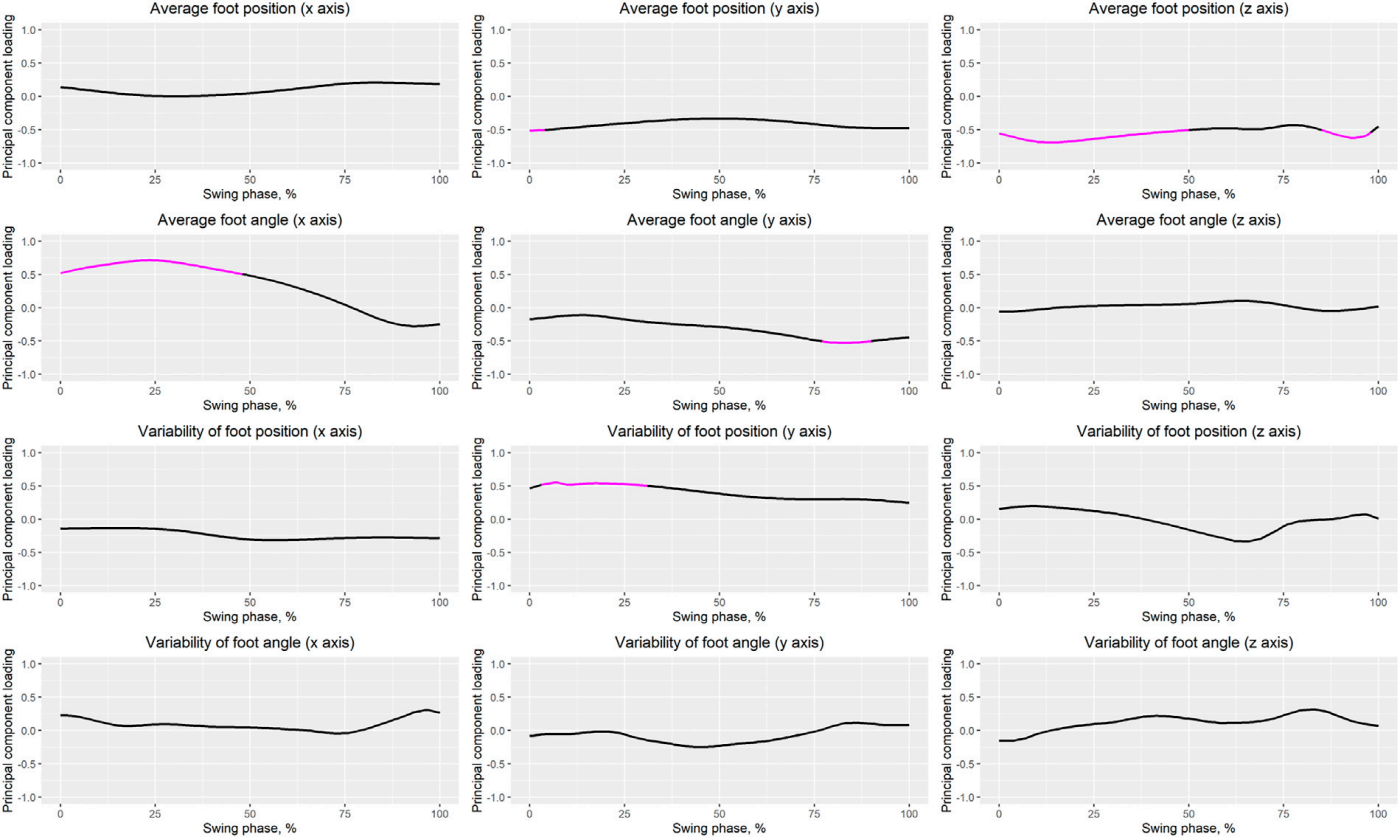
Waveforms of PCLs. The magenta solid line indicates that the absolute value of the PCL is greater than 0.5.

Table 1 presents the PCA results. We extracted 12 PCVs and found that the PCS of PCV3 in fallers was significantly larger than that in non-fallers (*p* = 0.003, *d* = 0.8 [large]). Figure 3 presents the reconstructed foot position and angle waveforms during the swing phase related to PCV3; Figure 4 presents the waveforms of the PCLs corresponding to PCV3. The absolute values of the PCL of PCV3 were larger than 0.5 at 0%–4% of the average foot position (*y*-axis), 0%–50% and 85%–98% of the average foot position (*z*-axis), 0%–48% of the average foot angle (*x*-axis), 77%–90% of the average foot angle (*y*-axis), and 3%–31% of the variability of foot position (*y*-axis). Based on these results, the main findings are that fallers have a 1) low average foot position on the *z*-axis (i.e., height) during the initial swing phase, 2) small average foot angle on the *x*-axis (i.e., rotation in the sagittal plane), during the initial swing phase, and 3) large variability in foot position on the *y*-axis (i.e., anterior/posterior position) during the initial swing phase compared to non-fallers. In other words, we can interpret these findings as follows: 1) foot height from the floor during the initial swing phase in fallers is low, 2) the height difference between the heel and toe during the initial swing phase in fallers is low (i.e., the rotation angle in the sagittal plane is small), and 3) variability of foot anterior/posterior position during the initial swing phase in fallers is large within our trials.

**TABLE 1:** Results of PCA.

	PCV1c		PCV2c		PCV3c		PCV4c	
Contribution rate, %	14.9		14.4		9.8		7.6	
Cumutative contribution rate, %	14.9		29.4		39.2		46.8	
Non-fallers, mean (SD)	0.08	(1.08)	−0.06	(1.01)	−0.37	(0.91)	0.00	(1.08)
Fallers, mean (SD)	−0.08	(0.92)	0.06	(1.00)	0.37	(0.96)	0.00	(0.93)
*p*-value	0.530		0.648		0.003		0.996	
d	0.16		0.12		0.80		0.00	

	PCV5c		PCV6c		PCV7c		PCV8c	
Contribution rate, %	6.7		5.8		5.1		4.7	
Cumutative contribution rate, %	53.5		59.3		64.3		69.0	
Non-fallers, mean (SD)	−0.12	(0.96)	0.11	(1.16)	0.03	(1.10)	0.18	(0.89)
Fallers, mean (SD)	0.12	(1.04)	−0.11	(0.82)	−0.03	(0.91)	−0.18	(1.08)
*p*-value	0.373		0.382		0.814		0.173	
d	0.23		0.23		0.06		0.36	

	PCV9b		PCV10c		PCV11c		PCV12b	
Contribution rate, %	3.8		3.1		2.5		2.3	
Cumutative contribution rate, %	72.8		75.9		78.4		80.7	
Non-fallers, mean (SD)	−0.05	(1.05)	−0.01	(1.00)	−0.11	(1.10)	−0.08	(0.96)
Fallers, mean (SD)	0.05	(0.96)	0.01	(1.02)	0.11	(0.90)	0.08	(1.05)
*p*-value	0.676		0.933		0.408		0.752	
d	0.10		0.02		0.22		0.16	

Note: a: Normality in only non-fallers was confirmed. b: Normality in only fallers was confirmed. c: Normalities in both groups were confirmed. d: Normalities were not confirmed in either group.

Table 2 presents the averages and variabilities of gait parameters for non-fallers and fallers. The averages of the maximum toe clearance, sagittal foot angle at toe-off, and peak sagittal foot angle in fallers were significantly lower, higher, and higher, respectively, compared to those in non-fallers. The variabilities of the timing of minimum toe clearance and timing of peak knee flexion angle in fallers were significantly larger compared to those in non-fallers.

**TABLE 2:** Averages and variabilities of gait parameters in non-fallers and fallers.

	Non-fallers		Fallers				
	Mean	(SD)	Mean	(SD)	*p*-value	d	Normality
Average							
Stride time, s	0.99	(0.09)	0.99	(0.08)	0.766	0.05	b
Stance time, s	0.58	(0.06)	0.59	(0.06)	0.625	0.04	b
Swing time, s	0.40	(0.03)	0.41	(0.03)	0.812	0.06	c
Cadence, steps/min	122.3	(10.0)	121.7	(10.1)	0.786	0.06	b
Gait speed, m/s	1.34	(0.16)	1.31	(0.15)	0.433	0.20	c
Stride length, m/HT	0.82	(0.06)	0.80	(0.06)	0.221	0.32	c
Step width, m/HT	0.050	(0.017)	0.042	(0.015)	0.055	0.48	b
Rate of stance phase, %	59.0	(1.3)	59.0	(1.3)	0.984	0.01	c
Maximum toe clearance (MaxTC), mm/HT	53.8	(6.8)	49.7	(5.6)	0.014	0.65	c
Minimum toe clearance (MinTC), mm/HT	33.1	(3.4)	31.7	(4.3)	0.148	0.38	c
Timing of MaxTC, %	25.0	(2.9)	24.3	(3.0)	0.376	0.23	c
Timing of MinTC, %	60.3	(5.3)	59.9	(3.8)	0.738	0.09	c
Sagittal foot angle at toe-off, °	−67.9	(6.9)	−64.0	(6.8)	0.030	0.57	c
Peak sagittal foot angle, °	−75.1	(7.1)	−70.9	(6.2)	0.018	0.63	c
Sagittal foot angle at peak knee flexion angle,°	−54.8	(4.7)	−53.1	(4.9)	0.171	0.36	c
Sagittal foot angle at heel contact, °	18.7	(3.8)	18.5	(3.6)	0.870	0.04	c
Timing of peak sagittal foot angle, %	9.5	(2.6)	9.5	(1.7)	0.981	0.01	c
Timing of peak knee flexion angle, %	30.8	(3.4)	30.2	(3.7)	0.494	0.18	c
Variability							
Stride time, s	0.014	(0.008)	0.014	(0.005)	0.542	0.05	b
Stance time, s	0.011	(0.005)	0.010	(0.004)	0.939	0.14	b
Swing time, s	0.009	(0.005)	0.008	(0.004)	0.711	0.09	d
Cadence, steps/min	1.70	(0.67)	1.72	(0.69)	0.931	0.02	c
Gait speed, m/s	0.030	(0.012)	0.032	(0.013)	0.610	0.13	c
Stride length, m/HT	0.014	(0.008)	0.016	(0.007)	0.080	0.35	b
Step width, m/HT	0.011	(0.005)	0.010	(0.004)	0.555	0.15	c
Rate of stance phase, %	0.65	(0.31)	0.61	(0.26)	0.959	0.12	b
Maximum toe clearance (MaxTC), m/HT	2.03	(1.02)	1.92	(0.86)	0.854	0.12	b
Minimum toe clearance (MinTC), m/HT	1.58	(0.89)	1.63	(0.75)	0.513	0.06	d
Timing of MaxTC, %	1.20	(0.40)	1.50	(0.85)	0.239	0.47	a
Timing of MinTC, %	1.76	(0.77)	2.19	(0.85)	0.044	0.53	c
Sagittal foot angle at toe-off, °	2.38	(1.16)	2.15	(1.14)	0.449	0.19	a
Peak sagittal foot angle, °	1.96	(1.25)	2.20	(1.15)	0.307	0.20	d
Sagittal foot angle at peak knee flexion angle,°	1.63	(0.71)	1.62	(0.68)	0.878	0.01	b
Sagittal foot angle at heel contact, °	1.01	(0.38)	1.04	(0.44)	0.765	0.08	c
Timing of peak sagittal foot angle, %	0.99	(0.50)	0.87	(0.42)	0.392	0.26	d
Timing of peak knee flexion angle, %	1.08	(0.46)	1.44	(0.67)	0.019	0.63	c

Note: Positive values of sagittal foot angle during the swing phase indicates that toe height is higher than heel height. a: Normality in only non-fallers was confirmed. b: Normality in only fallers was confirmed. c: Normalities in both groups were confirmed. d: Normalities were not confirmed in either group.

Table 3 presents the kinematics of non-fallers and fallers. The knee flexion angles at maximum toe clearance and peak knee flexion angle in fallers were significantly smaller compared to those in non-fallers. The hip flexion angle at the minimum toe clearance in fallers was significantly smaller compared to that in non-fallers. Table 4 presents the results of a regression analysis.

**TABLE 3:** Results of kinematics for non-fallers and fallers.

	Non-fallers		Fallers				
	Mean	(SD)	Mean	(SD)	p-value	d	Normality
Average							
At toe-off							
Posterior pelvic-tilt angle	-11.7	(5.9)	-9.9	(6.2)	0.249	0.30	c
Hip flexion angle	1.9	(9.4)	0.0	(9.3)	0.417	0.21	c
Knee flexion angle	40.8	(5.7)	40.6	(6.5)	0.866	0.04	c
Ankle dorsiflexion angle	-18.8	(6.6)	-15.8	(5.7)	0.059	0.50	c
At peak sagittal foot angle							
Posterior pelvic-tilt angle	-11.6	(5.9)	-9.7	(6.1)	0.234	0.31	c
Hip flexion angle	9.6	(9.6)	7.3	(8.9)	0.326	0.26	c
Knee flexion angle	54.0	(5.7)	53.0	(5.3)	0.490	0.18	c
Ankle dorsiflexion angle	-20.7	(7.4)	-17.6	(5.9)	0.074	0.47	c
At maximum toe clearance							
Posterior pelvic-tilt angle	-12.0	(5.9)	-10.0	(5.9)	0.190	0.34	c
Hip flexion angle	23.3	(7.3)	19.8	(8.0)	0.083	0.45	c
Knee flexion angle	66.4	(4.0)	63.8	(4.1)	0.018	0.63	c
Ankle dorsiflexion angle	-8.6	(5.1)	-8.0	(4.9)	0.645	0.12	c
At peak knee flexion angle							
Posterior pelvic-tilt angle	-12.1	(5.9)	-10.0	(5.9)	0.179	0.35	c
Hip flexion angle	28.0	(7.3)	24.6	(6.9)	0.064	0.49	c
Knee flexion angle	67.4	(4.2)	65.0	(3.9)	022	0.61	c
Ankle dorsiflexion angle	-4.7	(4.5)	-4.3	(4.1)	0.719	0.09	c
At minimum toe clearance							
Posterior pelvic-tilt angle	-12.5	(5.6)	-10.5	(6.0)	0.180	0.35	c
Hip flexion angle	42.7	(6.9)	38.9	(7.0)	0.042	0.54	c
Knee flexion angle	45.1	(4.5)	43.3	(3.4)	0.086	0.45	c
Ankle dorsiflexion angle	4.6	(3.5)	4.9	(3.1)	0.675	0.11	c
At heel contact							
Posterior pelvic-tilt angle	-12.3	(5.6)	-10.8	(6.0)	0.328	0.25	c
Hip flexion angle	40.4	(7.6)	37.7	(7.0)	0.157	0.37	c
Knee flexion angle	11.8	(4.6)	10.9	(3.2)	0.412	0.21	c
Ankle dorsiflexion angle	1.0	(3.3)	1.3	(3.1)	0.889	0.09	b

Note: Positive values of posterior pelvic-tilt, hip flexion, knee flexion, and ankle dorsiflexion angles indicate posterior pelvic-tilt, hip flexion, knee flexion, and ankle dorsiflexion, respectively. a: Normality in only non-fallers was confirmed. b: Normality in only fallers was confirmed. c: Normalities in both groups were confirmed. d: Normalities were not confirmed in either group.

**TABLE 4:** Results of regression analysis.

		Independent variable										
		Hip flexion angle			Knee flexion angle			Ankle dorsiflexion angle				
Model	Dependent variable	β	t value	*p*-value	β	t value	*p*-value	β	t value	*p*-value	Adjusted R2	*p*-value
1	Maximum toe clearance	−0.26	−1.9	0.066	0.63	4.6	0.000	0.15	1.3	0.196	0.26	0.000
2	Minimum toe clearance	−0.04	−0.2	0.809	0.23	1.6	0.113	0.06	0.5	0.653	0.00	0.363
3	Sagittal foot angle at toe-off	0.12	1.9	0.063	−0.31	−4.7	0.000	0.92	20.2	0.000	0.88	0.000
4	Peak sagittal foot angle	0.16	2.2	0.034	−0.29	−3.9	0.000	0.94	19.1	0.000	0.86	0.000
5	Sagittal foot angle at peak knee flexion angle	0.06	0.8	0.457	−0.41	−5.5	0.000	0.74	11.2	0.000	0.77	0.000
6	Sagittal foot angle at heel contact	0.22	2.7	0.009	−0.57	−7.0	0.000	0.74	10.5	0.000	0.71	0.000

Note: β indicates standard partial regression coefficient. Positive values of hip flexion, knee flexion, and ankle dorsiflexion angles indicate hip flexion, knee flexion, and ankle dorsiflexion, respectively.

Table 5 presents correlation coefficients between the PCSs of PCV3 and gait parameters. There were significant positive correlations between PCV3 and the averages of stride time, stance time, sagittal foot angle at toe-off, peak sagittal foot angle, and sagittal foot angle at peak knee flexion angle. However, there were significant negative correlations between PCV3 and the averages of gait speed, stride length, maximum and minimum toe clearances, and timing of peak knee flexion angle. There were significant positive correlations between PCV3 and variabilities of stride length, timing of minimum toe clearance, and sagittal foot angle at heel contact. Finally, there was a significant negative correlation between PCV3 and variability of minimum toe clearance.

**TABLE 5:** Results for correleation coefficients between gait parameters and PCV3.

	r	*p*-value
Average		
Stride time, s	0.26	0.043
Stance time, s	0.28	0.033
Swing time, s	0.21	0.112
Cadence, steps/min	−0.25	0.059
Gait speed, m/s	−0.44	0.000
Stride length, m/HT	−0.52	0.000
Step width, m/HT	−0.16	0.212
Rate of stance phase, %	0.20	0.123
Maximum toe clearance (MaxTC), m/HT	−0.47	0.000
Minimum toe clearance (MinTC), m/HT	−0.43	0.001
Timing of MaxTC, %	−0.09	0.480
Timing of MinTC, %	−0.17	0.190
Sagittal foot angle at toe-off	0.47	0.000
Peak sagittal foot angle	0.59	0.000
Sagittal foot angle at peak knee flexion angle	0.48	0.000
Sagittal foot angle at heel contact	−0.24	0.061
Timing of peak sagittal foot angle, %	−0.16	0.210
Timing of peak knee flexion angle, %	−0.36	0.005
Variability		
Stride time, s	0.19	0.149
Stance time, s	0.21	0.105
Swing time, s	0.20	0.131
Cadence, steps/min	0.12	0.350
Gait speed, m/s	0.04	0.766
Stride length, m/HT	0.31	0.015
Step width, m/HT	−0.24	0.068
Rate of stance phase, %	0.23	0.072
Maximum toe clearance (MaxTC), m/HT	−0.01	0.920
Minimum toe clearance (MinTC), m/HT	−0.34	0.007
Timing of MaxTC, %	−0.02	0.860
Timing of MinTC, %	0.27	0.036
Sagittal foot angle at toe-off	0.21	0.103
Peak sagittal foot angle	0.22	0.097
Sagittal foot angle at peak knee flexion angle	0.20	0.131
Sagittal foot angle at heel contact	0.27	0.038
Timing of peak sagittal foot angle, %	−0.02	0.853
Timing of peak knee flexion angle, %	0.23	0.075

## 4 Discussion

### 4.1 Main findings

The goal of this study was to identify the comprehensive characteristics of foot positions and angles during the swing phase in fallers using PCA. We found that the PCS of PCV3 in fallers was significantly larger than that in non-fallers (*p* < 0.003, Cohen’s d = 0.80 [large]). Therefore, we consider that the foot positions and angles during the swing phase related to PCV3 are important for assessing fall risk. Specifically, our main findings based on the reconstructed waveforms related to PCV3 (Figure 3) and PCL waveforms (Figure 4) can be summarized as follows. Compared to non-fallers, fallers had a 1) low average foot position on the *z*-axis (i.e., height) during the initial swing phase, 2) small average foot angle on the *x*-axis (i.e., rotation in the sagittal plane), during the initial swing phase, and 3) large variability in foot position on the *y*-axis (i.e., anterior/posterior position) during the initial swing phase. Therefore, we determined that PCV3 is the main PCV that reflects changes in foot positions and angles during the initial swing phase. Specifically, we define “changes in foot positions and angles in the sagittal plane during the initial swing phase” as our interpretation of PCV3.

### 4.2 Average foot position on the *z*-axis (foot height)

As shown in Figure 4, we observed that the absolute values of the PCL of the foot position on the *z*-axis in the initial swing phase (ranging from approximately 10%–20% of the swing phase) were especially large. Furthermore, based on Figure 3, we can conclude that the foot height during the initial swing phase is lower in fallers than in non-fallers. Many previous studies have focused on minimum toe clearance (i.e., foot height) during the mid-swing phase in fallers and non-fallers (Chiba et al., 2005; Khandoker et al., 2008b, 2008a; Barrett et al., 2010; Karmakar et al., 2013; Cebolla et al., 2015; Watanabe, 2018; Delfi et al., 2021). However, based on our results, we consider it important to focus on foot height during the initial swing phase (e.g., maximum toe clearance) to assess fall risk instead of focusing on foot height during the mid-swing phase. The maximum toe clearance observed in the initial swing phase in fallers was significantly lower than that in non-fallers (Table 2), but there was no significant difference in the minimum toe clearance observed in the mid-swing phase between groups, as shown in Table 2. Furthermore, the effect size of maximum toe clearance was larger than that of minimum toe clearance (0.65 and 0.38, respectively). Because PCA was performed in this study to identify the comprehensive characteristics of foot positions and angles during the entire swing phase, we were able to uncover this novel finding.

Based on Table 4 (Model 1), we found a significant positive relationship between the maximum toe clearance and knee flexion angle at maximum toe clearance. Furthermore, we confirmed that the knee flexion angle at maximum toe clearance in fallers was significantly smaller than that in non-fallers (Table 3). Based on these results, the first main finding can be attributed to differences in the knee joint angle between groups. We consider that the underlying reason for the significant difference in knee joint angle at maximum toe clearance observed in the initial swing phase is related to muscle weakness for hip flexion and ankle plantar flexion. According to a previous simulation study (Goldberg et al., 2004), activations of hip flexion and ankle plantar flexion muscles during the pre-swing phase cause large knee flexion angles during the swing phase. Furthermore, another study (Shin et al., 2012) reported that the strengths of the hip flexion and ankle plantar flexion muscles in fallers are lower than those in non-fallers. In this study, we did not measure strength for each participant, but this may be one of the reasons for the significant difference in the knee joint angle at maximum toe clearance between groups.

### 4.3 Average foot angle on the *x*-axis (rotation in the sagittal plane)

Based on Figure 4, regarding average foot angle on the *x*-axis, the absolute values of the PCL of PCV3 from 0% to 48% of the swing phase were larger than 0.5, and the absolute value of the PCL of PCV3 at approximately 25% of the swing phase (i.e., initial swing phase) was the largest. Based on Figure 3, the average foot angles on the *x*-axis during the initial swing phase in fallers were smaller than those in non-fallers, indicating that the average foot angle on the *x*-axis during this phase is important to distinguish fallers and non-fallers. In addition, we confirmed that the knee flexion angle at the peak knee flexion angle in fallers (occurring at 30.2% of the swing phase in Table 2) was significantly smaller than that in non-fallers (Table 3). Moreover, a significant negative relationship between the knee flexion angle and sagittal foot angle at peak knee flexion angle (Table 4; Model 5) was identified. Based on these results, we consider that the difference in knee joint angle during the initial swing phase between groups may be a reason for the small average foot angle on the *x*-axis.

### 4.4 Variability in foot position on the *y*-axis (anterior/posterior position)

We observed large variability in the foot position on the *y*-axis during the initial swing phase (specifically, from approximately 5%–30% of the swing phase; Figure 4) in fallers compared to non-fallers. Furthermore, we confirmed a significant positive relationship between PCV3 and stride length variability (Table 5). Based on these results, the variability in stride length may be affected by the variability in the foot position on the *y*-axis during the initial swing phase (i.e., from 3% to 31% of the swing phase). Many previous studies (Reelick et al., 2011; Doi et al., 2020; Bytyçi and Henein, 2021), have reported large variability in stride length in fallers compared to non-fallers (a trend of significant differences in variability in stride length was also observed in this study; Table 2). Therefore, variability in stride length is one of the indices used to assess fall risk, and we consider that our results may be useful for understanding the underlying reasons affecting the variability in stride length. Previous studies have reported that (1) appropriate movement of the center of mass from the pre-swing phase to the initial swing phase (i.e., from the double-limb support phase to the single-limb support phase) is necessary to maintain balance (Lugade et al., 2011), 2) activation of ankle plantar flexion during push-off is important for moving the center of mass forward (Neptune et al., 2008), and 3) the strength of the ankle plantar flexion muscles in fallers is lower than that in non-fallers (Shin et al., 2012). Based on these findings, we suppose that weakness of ankle plantar flexion muscles in fallers may be related to the large variability in the foot position on the *y*-axis during the initial swing phase.

### 4.5 Applications and limitations

Some previous studies have used insole devices with embedded inertial measurement units (Huang et al., 2021; Fukushi et al., 2022) or shoes with attached inertial measurement units (Mariani et al., 2010; Dadashi et al., 2013). It has been reported that an inertial measurement unit on the foot can calculate foot positions and angles during the swing phase (Mariani et al., 2010; Fukushi et al., 2022). Therefore, our findings regarding the characteristics of foot positions and angles during the swing phase in non-fallers and fallers may be beneficial for developing wearable devices that can evaluate fall risk using information collected during daily outdoor walking.

However, our study also has some limitations. First, all participants walked barefoot. It has been reported that footwear affects the kinematics of the ankle joint (Hida et al., 2021) and gait speed (Arnadottir and Mercer, 2000). Therefore, footwear (or a lack thereof) may have affected our main findings. Therefore, it is unknown whether our findings are applicable to walking with shoes. Second, all participants walked in a laboratory setting. A previous study (Takayanagi et al., 2019) reported that walking speed during daily walking is slower than that during laboratory walking. Another study reported that changes in gait speed affect the kinematics of the ankle joints during gait (Chehab et al., 2017). Therefore, it remains unclear whether our findings can be applied to daily walking. Based on these limitations, future studies should identify the characteristics of foot positions and angles during daily walking with shoes in non-fallers and fallers to assess fall risk using information collected during daily walking.

In conclusion, this study revealed the characteristics of foot position and angle related to fall risk using PCA. Our results indicated that fallers have a low average foot height, small foot angle in the sagittal plane, and large variability in foot anterior/posterior position during the initial swing phase compared to non-fallers. Our findings promote the understanding of the characteristics of older people with a high fall risk.

## Data Availability

The raw data supporting the conclusions of this article will be made available by the authors, without undue reservation.
